# The Missing Role of Prenatal Diagnosis of Chronic Histiocytic Intervillositis in the Management of Growth Restricted Fetuses

**DOI:** 10.3389/fmed.2021.809315

**Published:** 2022-02-02

**Authors:** Sedigheh Hantoushzadeh, Maasoumeh Saleh, Sepehr Aghajanian, Behnaz Nouri

**Affiliations:** ^1^Department of Obstetrics and Gynecology, Maternal-Fetal Neonatal Research Center, Valiasr Hospital, Tehran University of Medical Sciences, Tehran, Iran; ^2^Department of Obstetrics and Gynecology, Shariati Hospital, Tehran University of Medical Sciences, Tehran, Iran; ^3^Student Research Committee, School of Medicine, Alborz University of Medical Sciences, Karaj, Iran; ^4^Department of Community Medicine, School of Medicine, Alborz University of Medical Sciences, Karaj, Iran

**Keywords:** Doppler ultrasound, prenatal diagnosis, fetal growth restriction, chorionic villus sampling, chronic histiocytic intervillositis

## Introduction

Chronic histiocytic intervillositis (CHI) is a rare form of placental lesion with high fetal morbidity, mortality, and recurrence rate. It most commonly affects the terminal villi, the sites of gas, and the nutrient exchange, closest to the maternal surface. However, it is also frequently observed in other sites and may rarely present with diffuse involvement (1). The CHI is associated with adverse obstetric outcomes including recurrent miscarriage, fetal growth restriction (FGR) and impaired growth in 32% of the pregnancies reaching term, and intrauterine fetal death, with half of the miscarriages occurring at ≤ 12 weeks of gestation (2). The definite diagnosis is reliant on postnatal histopathological analysis and the lack of diagnostic biomarkers and consensus on diagnostic criteria that precludes accurate prenatal diagnosis (3). Nevertheless, FGR, oligohydramnious, and abnormal umbilical artery Doppler, especially in the presence of normal uterine arteries Doppler, and abnormal placental morphology in ultrasound can be associated with CHI. Normal uterine artery Doppler indices along with abnormal umbilical artery Doppler presentation strongly suggest that the sole cause of FGR could be linked to placental problems excluding malplacentation.

The majority of studies have provided recommendations on how to manage subsequent pregnancies, but the diagnostic value of CHI in current pregnancy and the management of FGR have not been described in the guidelines, thus, neglecting the possibility that the prenatal diagnosis of CHI could facilitate effective treatment options, optimal pregnancy care, and planning for termination of pregnancy, which could improve pregnancy outcomes.

## Diagnostic Approaches

The authors suggest that clinicians facing a case of FGR with unknown cause should consider seeking a diagnosis of CHI according to maternal obstetrical history, serum biomarkers and alkaline phosphatase (ALP), placental appearance, and Doppler indices in ultrasound. Placental MRI and chorionic villous sampling (CVS) could also aid in the diagnosis of CHI in early-onset severe FGR fetuses. However, due to the possibility of focal involvement, biopsies should be performed under the guide of ultrasound from placental areas that are apparently abnormal in imaging. Multiple biopsies may be needed to obtain samples from cases with small and restricted lesions. CVS is also useful for identifying FGR cases due to confined placental mosaicism. In conclusion, in the event of normal fetal, placental, umbilical cord, and uterine artery Doppler evaluation, and genetic and TORCH studies with unremarkable maternal history, physical examination, and laboratory studies, the aforementioned markers are beneficial in the diagnosis of CHI. Furthermore, histopathological analysis of placental markers as well as maternal immunological profile has also reported several findings which could be potentially utilized for antenatal diagnosis of CHI.

While fibrin deposition, trophoblast necrosis, and CD68+ M2-like macrophage infiltration have been mentioned as prominent histopathological factors of the postpartum placental biopsies (4), a limited number of studies have also mentioned increased maternal anti-HLA antibodies and cytotoxic T-lymphocyte precursor frequency, and CD39 downregulation in surface of villi and endothelial cells of the stem villi (5). Although these findings in preliminary studies may justify the experimental incorporation of the proposed maternal factors and CVS in select cases with high clinical suspicion, the diagnostic benefit of CVS should be judged against its widely recognized, yet, equivocally higher risk of miscarriage (6, 7).

## Therapeutic Interventions

Once the diagnosis is established, the FGR management can be performed accordingly. Although evidence regarding therapeutic interventions for CHI are few and far between, a small number of studies have suggested that thromboprophylactic drugs, such as aspirin and heparin, as well agents targeting the immune system, including hydroxychloroquine and glucocorticoids, may be of therapeutic benefit which is consistent with immunologic origin of the disease and the potential role of anti-human platelet antigen antigen-1a in induction of low-grade CHI (8, 9). For instance, hydroxychloroquine and prednisolone were associated with 62% improvement in live birth rate compared to untreated pregnancies (8). Furthermore, heterogenous combinations of all of the aforementioned agents in a multicenter study by Melkinian et al. led to the increased number of live births from 32 to 67%. Certain case reports have also provided indirect evidence of the effectiveness of these drugs by demonstrating successful pregnancy and birth with minimal placental changes in recurrent cases of CHI (10, 11). Notably, Ozawa et al. reported improved histologic findings using aspirin and prednisolone compared to aspirin alone and heparin plus aspirin in a case of three consecutive CHI pregnancies treated with each of the aforementioned regimens (10).

## Discussion

Although the lack of definite diagnostic markers has made the antenatal diagnosis of CHI rather difficult, the possibility of antenatal diagnosis and management of CHI is especially important in patients with a history of adverse pregnancy outcomes and in patients with diminished chance of achieving subsequent successful pregnancies due to aging, depleted ovarian reserve, infertility, or other maternal complications. In this regard, evaluating the specimen obtained during CVS of growth restricted fetuses with indications not necessarily related to CHI could provide means to further investigate cases with unknown diagnosis. Moreover, findings in placental ultrasound or MRI should also be re-evaluated and compared to placental histopathology after birth, especially in cases where no clear cause for FGR in the prenatal period has been identified. In line with this, determining CHI prevalence in early and late-onset FGR by placental evaluation after birth may further guide physicians in the management of FGR pregnancies in the future. Prenatal diagnosis of CHI offers the advantage of devising a potential management plan throughout the course of pregnancy, from fetal care and therapeutic interventions to effective termination of pregnancy. Nevertheless, further studies are needed on the combined use of the modalities mentioned throughout the text to assess their diagnostic accuracy, as well as randomized trials directed to examine the efficacy of current promising interventions and other immunomodulatory drugs in reaching the optimal outcome in identified pregnancies.

## Author Contributions

SH conceptualized and conceived the opinion provided in the text and edited the manuscript. MS and SA wrote the manuscript and contributed with data curation. BN contributed with data collection and editing of the manuscript.

## Conflict of Interest

The authors declare that the research was conducted in the absence of any commercial or financial relationships that could be construed as a potential conflict of interest.

## Publisher's Note

All claims expressed in this article are solely those of the authors and do not necessarily represent those of their affiliated organizations, or those of the publisher, the editors and the reviewers. Any product that may be evaluated in this article, or claim that may be made by its manufacturer, is not guaranteed or endorsed by the publisher.
